# How to create a balanced eye team: an example from Malawi

**Published:** 2018-07-31

**Authors:** Khumbo Kalua

**Affiliations:** 1 Director: Blantyre Institute for Community Outreach (BICO), & Professor, Lions Sight First Eye Hospital, PO Box E180, Blantyre, Malawi.

Malawi has a population of 17 million people, 87% of whom live in rural areas. There just twelve ophthalmologists in the whole country, equating to 1.4 ophthalmologists per million people – far short of the 4 per million recommended by the World Health Organization.^1^ To make matters worse, eleven of the ophthalmologists are based in urban areas.

Eye teams have aimed to address this by running outreach services that include cataract surgery. However, sustaining outreach services is expensive and requires a lot of travelling, which can disrupt the entire health system. Patients often have to wait for months before gaining access to eye care services. In our experience, staff fatigue eventually results in fewer visits being conducted.

To bridge the gap in eye services, Malawi's ministry of health has now established a task shifting approach. This means that some of the services usually offered by ophthalmologists are shifted to, and offered by, mid-level or allied eye health personnel who are recruited from rural hospitals and given additional training.

**Ophthalmic clinical officers (OCOs)** are clinical officers who have undergone training in ophthalmology and received a diploma. OCOs can manage most eye conditions and perform basic extraocular surgery. There is currently at least one OCO in each rural hospital. OCOs can train further and become cataract surgeons or trichiasis surgeons.

**Cataract surgeons** are OCOs who have attended an additional one-year surgical training course at a teaching hospital. They are able to perform cataract surgery within their rural hospital. Trainees remain on full salary, and a non-governmental organisation (NGO) pays for their training and upkeep.

**Trichiasis surgeons.** OCO's working in trachoma-endemic regions are trained and equipped to undertake trichiasis surgery at community level. They are usually trained ‘on the job’ over a period of two weeks.

**Optometrists/optometrist technicians** Optometrists have been trained Malawi since 2008 and an integral part of the eye care workforce, to provide basic ocular disease management and refractive services. They are active in both the private and government facilities. Optometry technicians are posted to rural hospitals, where they provide refractive services.

**Ophthalmic nurses** are general nurses who have received further training in eye care and are able to manage patients in the wards and assist in theatre.

**Figure:**
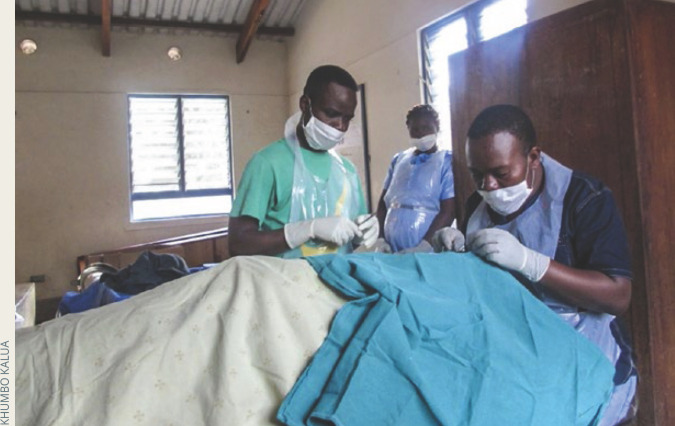
Task shifting: a trachoma trichiasis surgeon performing trichiasis surgery in the community. MALAWI

Equipment and consumables are often provided by the eye NGO partner working in an area, and supervision is conducted by the Regional Ophthalmologist in each area. For OCOs and cataract surgeons in particular, greater opportunities for promotion, and allowances for outreach work, encourage them to remain in the rural areas.

## What has been the impact of the task shifting programme?

- Eye service delivery has improved, resulting in fewer cases referred to a tertiary hospital. In the case of trichiasis surgery, there have been an average of over 1,000 operations per year over the last three years, compared to less than 200 per year before task shifting was introduced. Districts with cataract surgeons are managing almost all cataract operations (except for complicated cataract) at district level
- Additional training has increased the performance of mid-level/allied ophthalmic personnel
- Fewer staff members are now involved in outreach
- Activities, such as referrals between rural and urban eye units, are now better organised
- The geographical coverage of eye health services have increased
- There has been a reduction in the level of inequity in access to eye health services between rural and urban areas.

In conclusion, task shifting has addressed some of the human resource gaps in eye health in Malawi, and has helped improve service delivery, especially the delivery of trichiasis surgery.
